# Anaesthesia Monitoring by Recurrence Quantification Analysis of EEG Data

**DOI:** 10.1371/journal.pone.0008876

**Published:** 2010-01-26

**Authors:** Klaus Becker, Gerhard Schneider, Matthias Eder, Andreas Ranft, Eberhard F. Kochs, Walter Zieglgänsberger, Hans-Ulrich Dodt

**Affiliations:** 1 Bioelectronics, Vienna University of Technology, Vienna, Austria; 2 Department of Anaesthesiology, Klinikum rechts der Isar, Technische Universität München, Munich, Germany; 3 Neuronal Network Dynamics, Max Planck Institute of Psychiatry, München, Germany; Mount Sinai School of Medicine, United States of America

## Abstract

Appropriate monitoring of the depth of anaesthesia is crucial to prevent deleterious effects of insufficient anaesthesia on surgical patients. Since cardiovascular parameters and motor response testing may fail to display awareness during surgery, attempts are made to utilise alterations in brain activity as reliable markers of the anaesthetic state. Here we present a novel, promising approach for anaesthesia monitoring, basing on recurrence quantification analysis (RQA) of EEG recordings. This nonlinear time series analysis technique separates consciousness from unconsciousness during both remifentanil/sevoflurane and remifentanil/propofol anaesthesia with an overall prediction probability of more than 85%, when applied to spontaneous one-channel EEG activity in surgical patients.

## Introduction

In today's clinical practice, routine monitoring of general anaesthesia is based mainly on cardiovascular parameters and motor responses. If surgical stimulation provokes neither movement, nor an increase in heart rate or blood pressure, it is assumed that the anaesthesia is sufficient. However, during neuromuscular blockade, in the presence of beta-blockers, or in patients who only tolerate ‘light levels’ of anaesthesia, these clinical parameters may fail to reliably monitor the depth of anaesthesia. Despite the stability of these parameters, patients may become conscious during surgery, potentially leading to explicit memory of words spoken in the operating room, discomfort, or pain. In addition, if the central processing of stimuli is not sufficiently blocked, implicit memories may be acquired via auditory or other sensory input under general anaesthesia. Possible consequences of intraoperative awareness include nightmares, or even symptoms of a posttraumatic stress disorder [1].

As recently suggested by us [2], [3] and others [4], RQA of EEG recordings appears to be a highly promising tool for monitoring the depth of anaesthesia. One of the basic requirements for monitoring the hypnotic level of anaesthesia is the ability to separate consciousness from unconsciousness. The present analysis was performed to assess the ability of RQA to separate between consciousness and unconsciousness at the transition between these clinical stages.

## Methods

RQA was applied to EEG data from two clinical studies comprising 40 patients each (study I and study II). In both studies, patients were randomly assigned to receive either remifentanil/sevoflurane or remifentanil/propofol anaesthesia. In 30 s intervals, patients were asked to squeeze the investigator's hand. Anaesthesia was slowly induced until patients stopped following this command (first loss of consciousness, LOC 1). Subsequently, anaesthetic concentrations were increased to reach an appropriate level of anaesthesia for intubation. The isolated forearm technique [5] was used to maintain the patient's ability to follow commands, and succinylcholine was given to facilitate endotracheal intubation. After intubation, propofol or sevoflurane administration was stopped until the patients followed commands again (first return of consciousness, ROC 1). Propofol or sevoflurane concentrations were increased, until the patients stopped squeezing the hand (second loss of consciousness, LOC 2), and surgery was performed. At the end of the surgical procedure, remifentanil and sevoflurane or propofol were discontinued and patients were assessed again for their ability to squeeze a hand on command (second return of consciousness, ROC 2).

A single-channel EEG was recorded from an electrode at the left temporal region (between the lateral edge of the eye and the upper edge of the ear, AT1) and referenced at Fpz (with the ground electrode at Fp1). The sampling frequency of the EEG data in study I was 250 Hz. No low pass was used and the notch filter (50 Hz) was enabled. The sampling frequency of the data in study II was 1 kHz, digitized with 12 bit amplitude resolution. To achieve equal sample rates, the EEG data from study II were resampled offline at 250 Hz, after low-pass filtering at 125 Hz. From both studies, data were selected from LOC (1 and 2) as well as from ROC (1 and 2) for analysis. All analyzed data segments were 30 s long, comprising the last 30 s before the last unanswered command (LOC, unconsciousness), and the first 30 s after the first answer to command (ROC, consciousness), respectively.

### EEG Data Analysis

The recorded EEGs were analyzed by RQA. RQA is a nonlinear time series analysis technique developed by J. P. Zbilut and C. Webber [6] on the basis of previous research from J. P. Eckmann and colleagues [7]. RQA quantifies the number and duration of recurrences of a dynamical system presented by its phase space trajectory. Compared to other techniques of nonlinear signal analysis it has the advantage that it requires no assumptions, on stationarity or linearity, concerning the analyzed data [8]. Amongst other parameters, RQA evaluates the determinism *D* of a time series, which has been shown to be a highly sensitive measure of predictability (regularity) [6], [8]. Mathematically, *D* is defined as the percentage of recurrence points, which are placed along diagonal lines of minimal length *l*_min_ in the recurrence plot of the investigated dynamical system. A value of *D* approaching 100% indicates almost perfect predictability, e.g. a straight line; a value approaching 0% indicates the absence of almost any predictability, e.g. Gaussian white noise. For EEG analysis we defined the complexity *C* as an inverse function of *D:*

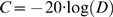
(1)


**Figure 1:**
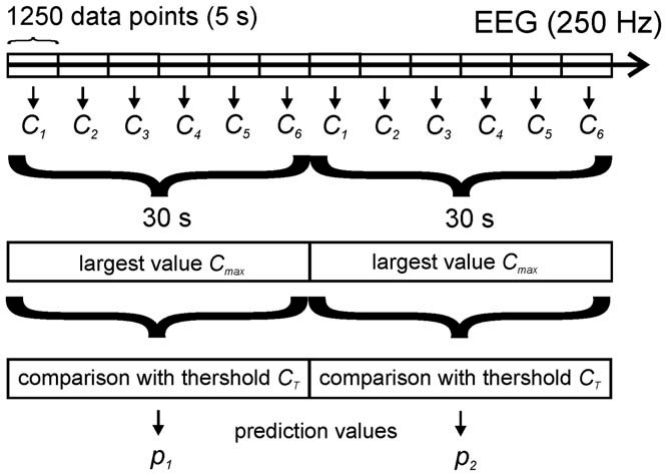
Principle of analysis. 5 s long successive data segments are analyzed by RQA yielding complexity values *C_1_*…*C_n_.* From 6 consecutive *C_i_'s,* the largest values C_max_ are compared with a threshold C_T_ to generate the decision values *p_i_* for making a conscious/unconscious decision.

According to (1) high values of *C* indicate that an EEG is highly irregular (‘chaotic’), low values indicate that it contains a certain amount of repetitive structures and thus is predictable to a certain extent. We performed RQA in non-overlapping time windows, each 5 s (1250 sample points) wide (windowed RQA [8]). From six consecutively calculated values of C we only used the largest value C_max_ for generating a single prediction value by comparison of C_max_ with a fixed threshold C_T_. Thus, one prediction value *p* was generated per 30 s interval (**Figure 1**).

RQA requires the specification of four preset parameters: a) An appropriate embedding dimension *m* b) A time lag *τ* for appropriate time-delay embedding of the EEG data according to Taken's theorem [9]. c) A value *r_max_*, describing the radius of the sphere in which radius neighboured phase space locations are assumed to be recurrent. *r_max_* was always scaled to the highest occurring distance in phase space for the current data segment. d) The minimal length *l_min_* of the left-upward diagonals in the recurrence diagram were taken into account for the calculation of *D* [6]. Unlike Zbilut and Webber [6], we used a Manhattan (City-block) metric for calculating phase space distances, i.e. the phase space distances were calculated as:
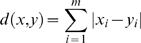
(2)


*d*: distance between to vectors *x* and *y*, *m*: embedding dimension.

Compared to Euclidean metrics (L_2_-norm), city-block metric (L_1_-norm) reveals more information about the local behaviour of recurrences [10]. Furthermore, it is computationally more efficient, whilst being more robust in its significance, with respect to an increase in recurrence numbers [10].

### Optimization of RQA Parameters-Assessment of RQA Results

We employed a box bounded global mixed-integer optimization algorithm [11] to determine best possible values for the RQA parameters *m*, *τ, r_max_,* and *l_min_* with respect to an optimal prediction probability *p_k_* for the ‘awake’ state using a training procedure. Mixed integer optimization uses a special algorithm, which enables the optimization variables to be fixed to discrete values as is required for the embedding dimension *m*. *P_k_* describes the correlation between an anaesthetic depth indicator value and observed anaesthetic depth. It has a value of 1 when the indicator predicts observed anaesthetic depth perfectly and a value of 0.5 when the indicator predicts no better than a 50∶50 chance [12]. For two-state systems, such as the regarded conscious/unconscious decisions, *p_k_* is equal to the area under the receiver-operator characteristics (roc) curve [12]–[13]. The numerical parameter range in which numerical optimization was performed was 1 to 25 for *m*, 4 to 200 ms for *τ,* 1 to 75% for *r_max_*, and 4 to 400 ms for *l_min_*. For training the algorithm, we used five different data subsets taken from study 1. Each of the training data subsets comprised one randomly selected third of the ROC events contained in study. The length of the training segments always was 30 s, comprising the values from the last 30 s before the last unanswered command (LOC, unconsciousness), and the first 30 s after the first answer to command (ROC, consciousness), respectively. The parameter values obtained in the five optimization runs varied between 2 and 6 for *m*, between 4 ms and 20 ms for *τ*, between 55% and 70% for *r_max_*, and between 12 ms and 32 ms for *l_min_*.

As two signals were analyzed at every clinical event LOC1, LOC2, ROC1, and ROC2, a maximum of 320 signals was available from studies I and II. The signals are situated either completely in a phase of consciousness or in a phase of unconsciousness. For study I, 282 signals and for study II 302 signals were used for pk analysis. Thus 38 signals were excluded from study I and 18 signals were excluded from study II. These signals contained artefacts expressed by signals of constant amplitude (flat line), values exceeding the measuring range of 250 µV, or were not of sufficient length.

## Results

### Training of the Algorithm

**Table 1** shows the prediction probabilities obtained in the five training runs performed with different randomly selected data subsets taken from study 1. Obviously, the combination *m* = 3, *τ* = 16 ms, *r_max_* = 70%, and *l_min_* = 20 ms yielded the highest *p_k_* (0.86±0.023, errors are always specified as standard error with non-parametric assumptions), if applied to the entire study I, i.e. to all 282 considered LOC/ROC and ROC/LOC transitions (**Table 1**). We therefore decided to use this parameter set for all further evaluations of the ability of RQA to separate consciousness from unconsciousness by means of studies 1 and 2.

**Table 1:** Optimization of RQA parameters.

N	RQA parameters as determined in 5 training runs, each using one randomly selected third of the ROC's of study I.					*p_k_* for complete study I (ROC + LOC)
	*m*	*τ* [*ms*]	*r_max_* [%]	*l_min_* [ms]	*p_k_*	
1	**3**	**16**	**70**	**20**	**0.88**	**0.86**
2	3	4	66	24	1.0	0.84
3	6	4	55	12	0.87	0.82
4	2	20	70	20	0.96	0.84
5	3	4	57	32	0.91	0.83

RQA parameters were optimized using 5 sets of training data, each comprising one randomly selected third of the ROC events in study I each. The parameter combination used for further evaluation is printed in bold.

### Prediction Probabilities

**Figure 2:**
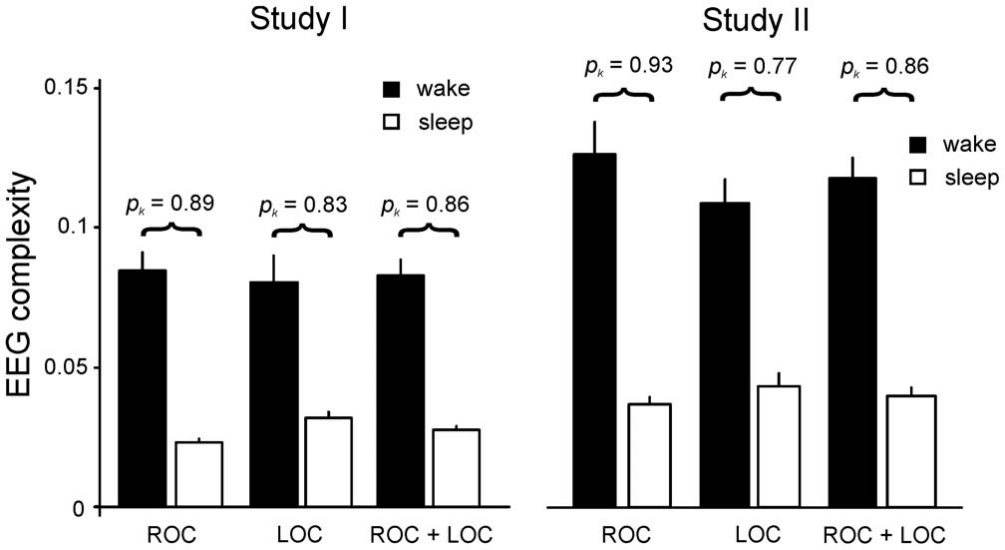
Complexities *C* and prediction probabilities *p_k_* obtained by RQA of the wake-sleep (LOC) or sleep-wake (ROC) transitions from studies I and II.

The achieved prediction probability for the complete study I was 0.86±0.023 (ROC + LOC). For this evaluation ROC and LOC transitions were pooled and one common *p_k_* was calculated. In two further calculations we considered only the ROC transitions or only the LOC transitions of the study, respectively. Considering only the ROC events resulted in a higher value (0.89±0.029), considering only the LOC events yielded a lower value (0.83±0.037) than that obtained if ROC and LOC events were pooled (**Figure 2** **)**.

Our results were verified by means of study II. For this evaluation no modifications to the algorithm or to the RQA parameters as determined from study 1 were made. We obtained a *p_k_* of 0.86±0.022 (ROC and LOC events), notably, this was identical to the *p_k_* obtained from study I. As in study I, ROC and LOC transitions were pooled and a common pk was obtained. Again, we found the *p_k_* was higher when considering only the ROC events (0.93±0.020) and lower (0.77±0.039) when considering only the LOC events (**Figure 2**).

Additionally, we tested the other four RQA parameter sets, determined in the five training runs (**Table 1**) by means of study II. Consistently, the *p_k_* values obtained with these parameter sets were all lower than the *p_k_* values obtained with the parameter set found optimal for study I (**Table 2**). Thus, the results obtained from study II are remarkably close to those obtained from study I. This clearly demonstrates that no over-fitting occurred during the training procedure.

**Table 2:** Verification of the algorithm by means of study II.

	*m*	*τ* [*ms*]	*r_max_* [%]	*l_min_* [ms]	*p_k_* (ROC + LOC)
1	**3**	**16**	**70**	**20**	**0.86**
2	3	4	66	24	0.78
3	6	4	55	12	0.81
4	2	20	70	20	0.82
5	3	4	57	32	0.81

As in study I the parameter combination *m* = 3, *τ* = 16 ms, *r_max_* = 70%, and *l_min_* = 20 ms gave the highest *p_k_*.

### Homogeneity of the Data

**Figure 3:**
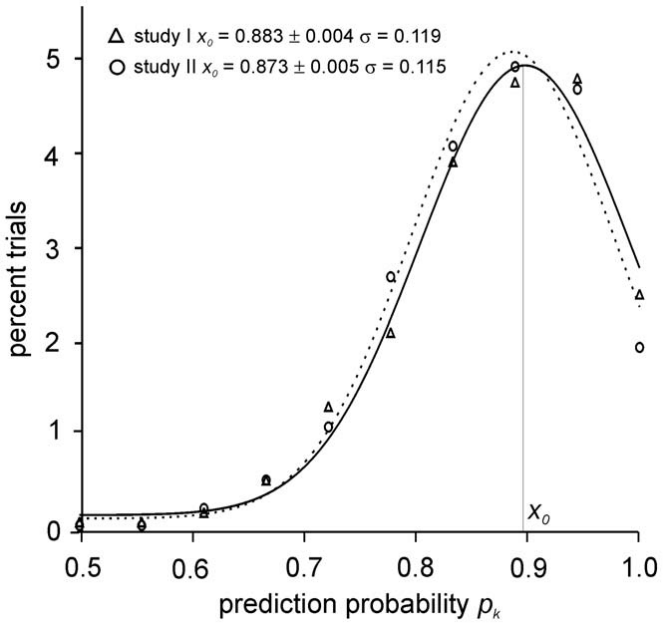
Homogeneity test. 1000 random samples comprising 10 ROC or LOC events each were drawn from studies I and II respectively. The figure shows a histogram of the *p_k_* values calculated from these subsamples.

To test if the quality of our EEG data is homogeneous, or if it might suffer from outliers, we repetitively drew random samples from studies I and II, consisting of 10 transition (ROC or LOC) events each. This procedure was repeated 1000 times, resulting in 10000 randomly composed data subsets for each study. For all of these subsets we determined the according *p_k_* values and binned the results into a histogram. As is obvious from **Figure 3**, the *p_k_* values were strictly normal distributed for both studies (*p* = 0.886±0.005 for study I and *p* = 0.890±0.004 for study II, Kolmogorov-Smirnov Test), suggesting that the quality of the EEG data in studies I and II is not critically suffering from outliers.

### Specificity and Sensitivity

**Figure 4:**
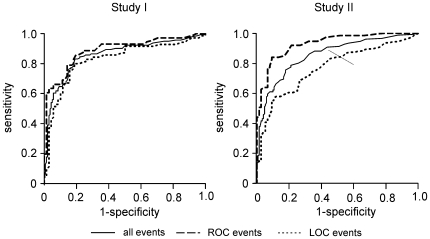
Roc-curves describing the relation between sensitivity and specificity of conscious/unconscious decisions using different threshold values C_T_.

To determine cut-off values *C_T_* for *C* which would allow a best possible decision between consciousness and unconsciousness, we calculated roc-curves for studies I and II, illustrating the relationship between sensitivity and specificity for given values of *C_T_* [13] (**Figure 4**, **Table 3**). Considering ROC and LOC events together, a sensitivity of about 0.90 is achieved with a specificity of about 0.56 (for study I, as well as for study II). For the ROC events alone, a sensitivity of 0.90 corresponds to a clearly improved specificity of about 0.68 (study I) or 0.8 (study II).

**Table 3:** Receiver-operator characteristics (ROC).

	Study I (ROC + LOC)		Study I (ROC)		Study I (LOC)	
*C_T_*	sensitivity	specificity	sensitivity	specificity	sensitivity	specificity
0.0265	0.92	0.35	0.93	0.38	0.91	0.33
0.0325	0.90	0.56	0.93	0.61	0.87	0.51
0.0355	0.88	0.65	0.90	0.68	0.86	0.61
0.0495	0.81	0.82	0.83	0.82	0.79	0.81
0.0625	0.71	0.85	0.72	0.86	0.70	0.84
0.0815	0.60	0.94	0.61	0.99	0.59	0.90
	**Study II (ROC + LOC)**		**Study II (ROC)**		**Study II (LOC)**	
*C_T_*	sensitivity	specificity	sensitivity	specificity	sensitivity	specificity
0.0265	0.86	0.68	0.89	0.80	0.83	0.55
0.0235	0.91	0.56	0.95	0.68	0.87	0.45
0.0350	0.70	0.83	0.77	0.92	0.62	0.75
0.0285	0.80	0.74	0.84	0.87	0.76	0.62
0.0335	0.71	0.83	0.79	0.92	0.63	0.75
0.0505	0.60	0.93	0.63	0.97	0.57	0.89

Sensitivity and related specificity for predicting the wake state at different cut-off values *C_T._*

## Discussion

Our results support the view that monitoring of the activity of the main target organ of general anaesthetics, i.e., the brain, may provide a method to assess the level of consciousness. The obtained *p_k_* value of >0.85 reflects an encouraging result for the analysed challenging data sets.

Selection of a 30 s period immediately preceding LOC or following ROC provides data, which are very close to each other in terms of not only time but also clinical status. As a consequence, we showed that RQA of the EEG is a method to separate ‘just unconscious’ from ‘just conscious’. The same data sets were presented to different monitors, the majority of which separated consciousness from unconsciousness with considerably lower *p_k_* values (0.5–0.8) [14]–[17]. So far, the best results have been obtained by the GE Entropy module, which analyses spectral entropy of the EEG, i.e., also a parameter of irregularity. As both entropy and RQA quantify the degree of regularity in the EEG signal, this may be a strong indication of an increase of EEG regularity as a general mechanism underlying anaesthesia-induced unconsciousness.

The cut-off values *C_T_* slightly differed between study I and II. A sensitivity of 0.9 corresponds to a *C_T_* of 0.0325 in study I, and to a *C_T_* of 0.0235 in study II (**Table 3**). This may be because the complexity values obtained from study II were generally larger than those obtained from study I (**Figure 2**). This is probably due to differences in the EEG-amplifiers that were used in the study.

A potential limitation of the present approach may be the selection of the maximum complexity value from a 30 s interval. If implemented in a monitor, this approach could produce a delay of up to 30 s before the state of consciousness is correctly indicated. This 30 s delay reflects a critical time interval. As Dutton et al. (1995) showed, the risk of postoperative recall increases, if patients are intraoperatively awake for more than 30 s. [18].

In this regard, consciousness (or wakefulness) during anaesthesia may be seen as an early warning sign of possible awareness with or without implicit or explicit recall. Therefore, the present study was designed to separate consciousness from unconsciousness. Consciousness was defined as an adequate response to a particular command. This reflects an intact working memory. The working (or short-term memory) spans a short time interval (approx. 30 s) and contains everything an individual thinks or perceives. After processing in the working memory, contents may either be forgotten or stored and form conscious (explicit) or unconscious (implicit) memory [19]. As a consequence, prevention of consciousness will prevent formation of both implicit and explicit memory.

Currently, available monitors require even longer time intervals before they reflect a change in the level of anaesthesia as indicated by changes of the EEG [16]. However, we expect that with higher sampled EEG data (1 kHz or above), combined with further improvements in the algorithm, shorter response times <10 s may become possible.

A potential limitation of the approach is that the analysis also includes high frequency components of the EEG signal. Particularly if electrodes are placed on the forehead, high frequency EEG signals may be contaminated with an electromyogram (EMG) of the frontal muscle, which is in the same frequency range and has higher amplitudes. As a consequence, analysis may be based on (unspecific) EMG rather than (specific) EEG. It has been shown for the EEG bispectral index (BIS), that with such an approach a patient who is fully awake but paralyzed may be classified as unconscious, because high frequency signals are diminished or blocked by neuromuscular blockade [20].

The selection of a maximum within a 30 s. interval may induce a bias towards higher values indicating ‘consciousness’. This may increase RQA values before LOC and after ROC (towards consciousness values). Conversely, results from “unconsciousness” should also be biased towards higher values in this case and, therefore, the overall statistical result should remain nearly unchanged.

Unfortunately, it is almost impossible to measure the brain concentration of an anaesthetic drug. Therefore, it is extremely difficult to test whether a parameter reflects a certain drug concentration in the brain. On the other hand, loss and return of consciousness are clinically relevant measures of the main effect of anaesthetic drugs, which can readily be assessed. Our study showed that RQA can be a useful measure of this main effect of general anaesthesia for both propofol and sevoflurane. In a test with two independently acquired EEG studies we achieved an overall prediction probability *p_k_* of >0.85. This value is considerable better than the *p_k_'s* obtained with devices being presently commercially distributed [14]–[15]. The described RQA-based algorithm is easily implemented on a modern personal computer in real-time. Thus, our study opens a new avenue for the development of improved future anaesthesia monitoring devices.
